# Numerical Methods for the Analysis of Power Transformer Tank Deformation and Rupture Due to Internal Arcing Faults

**DOI:** 10.1371/journal.pone.0133851

**Published:** 2015-07-31

**Authors:** Chenguang Yan, Zhiguo Hao, Song Zhang, Baohui Zhang, Tao Zheng

**Affiliations:** 1 State Key Laboratory of Electrical Insulation and Power Equipment, Xi’an Jiaotong University, Xi’an, China; 2 Alstom Grid, Redmond, Washington, United States of America; University Paul Sabatier, FRANCE

## Abstract

Power transformer rupture and fire resulting from an arcing fault inside the tank usually leads to significant security risks and serious economic loss. In order to reveal the essence of tank deformation or explosion, this paper presents a 3-D numerical computational tool to simulate the structural dynamic behavior due to overpressure inside transformer tank. To illustrate the effectiveness of the proposed method, a 17.3MJ and a 6.3MJ arcing fault were simulated on a real full-scale 360MVA/220kV oil-immersed transformer model, respectively. By employing the finite element method, the transformer internal overpressure distribution, wave propagation and von-Mises stress were solved. The numerical results indicate that the increase of pressure and mechanical stress distribution are non-uniform and the stress tends to concentrate on connecting parts of the tank as the fault time evolves. Given this feature, it becomes possible to reduce the risk of transformer tank rupture through limiting the fault energy and enhancing the mechanical strength of the local stress concentrative areas. The theoretical model and numerical simulation method proposed in this paper can be used as a substitute for risky and costly field tests in fault overpressure analysis and tank mitigation design of transformers.

## Introduction

Power transformers play a very critical role in electric energy transmission. A minor fault on it could seriously affect the reliability and stability of power system. However, oil-immersed transformers which contain large quantity of insulating oil are susceptible to internal arcing faults. Once the tank rupture or explosion occurs under overpressure due to the high-energy arcing faults, its internal combustible substance would spray to nearby staffs and power equipment and thus lead to disastrous consequences in personal, property and environmental safety.

According to the law of energy conservation, power energy of the faults converts into heat, kinetic and other forms of energy during internal arc duration. In 2009, the task force of IEEE power transformer subcommittee summarized the following [1]: the overpressure phenomenon inside transformer occurs when an internal arcing fault causes generation of large volume of decomposed gas; once the combined effect of the static overpressure and dynamic pressure wave propagation exceed the mechanical strength of the transformer tank, tank deformation or explosion is inevitable.

Being aware of the great hazard of transformer internal pressure which rises sharply when low-impedance faults occur, much theoretical and experimental effort on the tank overpressure, rupture and mitigation has been devoted since 1970s. The first significant contribution was presented by W. R. Mahieu in [2]. Mahieu assumed that the oil is incompressible, based on which the amplitude of pressure induced by an arc in a gas blanket located at the top of the tank was computed. In 1988, a more accurate method was invented by M. Foata, M. Iordanescu, and C. Hardy, who built a 2D potential flow model and used finite element method to study the dynamic response of transformer tanks under overpressure regardless of the fact that the oil is compressible and viscous [3]. Later in 2003, J. B. Dastous, A. Hamel, and M. Foata initiated a method using semi-experiment to calculate the overpressures generated by diverse arcs for different tank geometry parameters [4]. Recently in the CIGRE International conference 2010, they discussed and analyzed the major causes of tank rupture, tank containment capability and the performances of the protection devices[5].

As the computation capability advances, a 3-D numerical tool was presented in the study of complex physical phenomena after transformer internal faults [6]. The kernel of the numerical tool is based on a simplified 5-equation model proposed in [7], which was to describe the hydrodynamic behavior of the compressible 2-phase flows. In addition, physical effects such as electromagnetic forces, viscous, thermal and gravity effects were taken into account. In [6], it was assumed that the gas bubble is created instantaneously and the energy of the internal faults is used to entirely decompose the oil rather than increase the pressure. In fact, arc energy injects continually within the faults duration. The quantity and the internal pressure of gas bubble increase accordingly. As a result, there are obvious variances between their calculated and measured results in [6].

In 2010, a refined 3D numerical simulation method about mechanical effects of transformer internal faults was presented in [8]. With the assumption that the gas generation process from decomposition of the oil is adiabatic and the plates are modeled by shell elements without thickness, the paper simulated the fairly complex rupture of transformer tank, especially for the chimney, under overpressure caused by low-impedance arcing faults. Besides, A. Hackl and P. Hamberger put forward a static finite element analysis of a transformer tank under static overpressure with three material models [9]. Unfortunately, this work fails to solve the actual mechanical behaviors of tank wall due to the fact that the dynamic process of overpressure inside the tank was ignored. The previous investigations focus, including but not limited to the transformer tank rupture and mitigation, provides encouraging and representative results which are useful for tank structural design and fault overpressure analysis.

In order to study the transient characteristics of transformer tank under internal overpressure due to high-energy arcing faults, the whole process was considered as a particular acoustic and mechanical problem in this paper. Firstly, the basic models of transformer internal faults overpressure, tank mechanical deformation and acoustics-mechanical field coupling were proposed. Secondly, according to the practical geometric and nameplate parameters, a 3-D simulation model for the representative 360 MVA/220 kV power transformer was built as a study case. Besides, the transformer tank was considered as elastic-plastic body, described by a trilinear isotropic hardening model, for calculating accurately in our work. Thirdly, to illustrate the effectiveness of the proposed method, a 17.3MJ and a 6.3MJ arcing fault were assumed and the transformer internal overpressure distribution, wave propagation, von-Mises stresses distribution were solved by taking advantage of 3-D FEM. The simulation results show that there is a positive correlation between the mechanical stress and the overpressure amplitude, which is determined by the arc energy. Rapid fault clearance is an effective way to reduce the faults energy injection. Furthermore, both the pressure and mechanical stress distribution are far from being uniform inside the tank and the stress is always concentrated at some particular joints.

## Methods

### Transformer Internal Fault Overpressure Model

Generally, low impedance faults, such as turn-to-turn faults, are in the form of arc inside power transformer tanks once the oil or other insulating medium lose their dielectric properties. The energy generated by the arc is [10]:

$$W_{arc}=\int_{0}^{\Delta t}u_{arc}i_{arc}dt$$ (1)

where *W*_*arc*_ (kJ) is the total arc energy; *u*_*arc*_ (V) is the voltage drop across the arc; *i*_*arc*_ (kA) is the arc current; Δ*t* (s) is its duration.

In order to facilitate the integral of (1), it is assumed that the arc voltage *u*_*arc*_ is always positive and the arc current *i*_*arc*_ is changed into absolute value:

$$W_{arc}=\int_{0}^{\Delta t}u_{arc}\left|i_{arc}\right|dt$$ (2)

Regardless of the voltage drop near the electrode and extinguish peak of the arc, it is considered that the strength of electric field per unit length of arc column is constant during each current half wave [11]. In fact, the effective arc length is longer than the insulation distance in many cases and it is related to the pressure as well. According to [5, 12], a more accurate expression is recommended:

$$u_{arc}=55l_{arc}\sqrt{p/p_{0}}$$ (3)

where *l*_*arc*_ (m) is its length which is relevant to the fault severity and *p/p*_0_ represents the absolute pressure, the atmospheric pressure *p*_0_ equals to 101.3 kPa.

**Fig 1:**
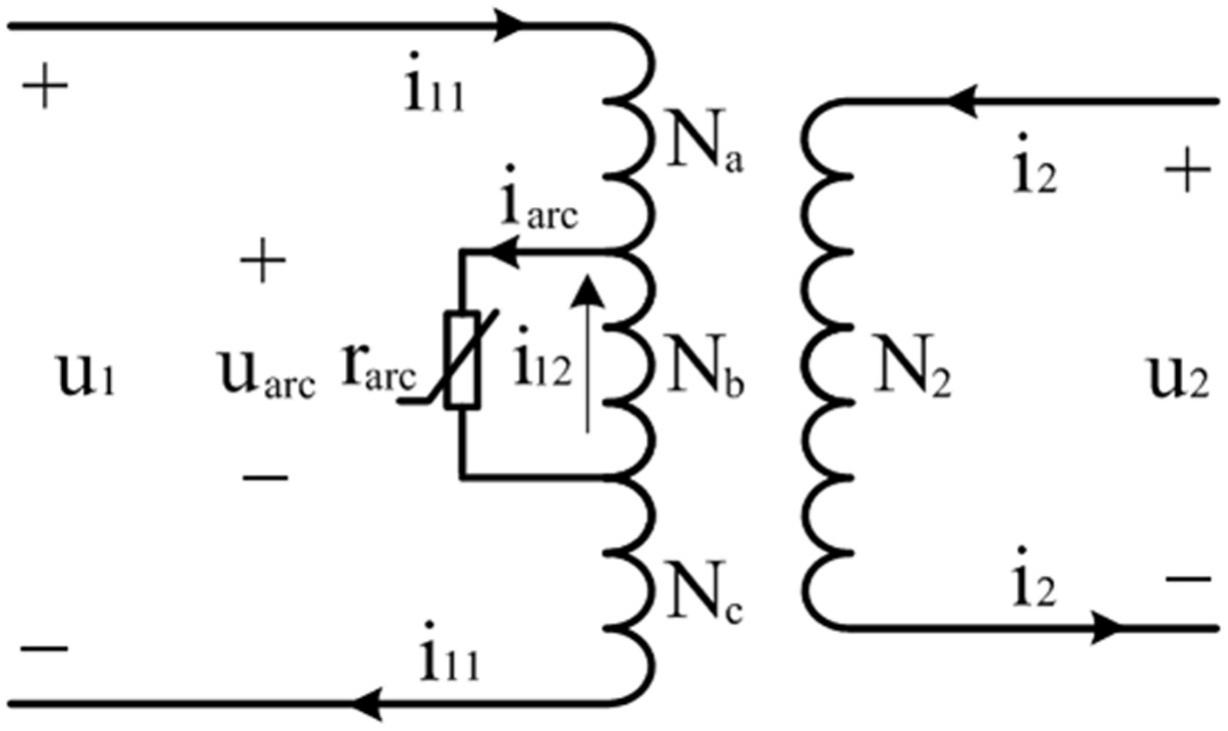
Two-winding transformer turn-to-turn short circuit at primary side.

In order to discuss the method to estimate the arc current, a single-phase two-winding transformer turn-to-turn arcing fault equivalent circuit was provided in Fig 1.

According to the Kirchhoff’s current law, the arc current *i*_*arc*_ is equal to the sum of primary side current *i*_11_ (kA) and inter-turn fault circulation *i*_12_ (kA). The transformer models presently being used of winding faults, have some deficiencies [13–15]. The differences among these models are the degree of approximation of reactance and inductance parameters. In this paper, the common method proposed in [13] is used to calculate the arc energy considering that its error is acceptable. Hence, the transformer turn-to-turn arcing fault energy can be calculated by

$$W_{arc}=55l_{arc}\int_{0}^{\Delta t}\sqrt{p/p_{0}}\left|i_{11}\text{+}i_{12}\right|dt$$ (4)

Based on the law of energy conservation, the released arc energy must be converted into energy loss, thermal transmission, chemical energy, mechanical energy and other forms [16]. Here, the energy to heat the gas bubble at constant volume can be denoted as *W*_*heat*_ (kJ) and its differential form is:

$$dW_{heat}=k_{heat}dW_{arc}=k_{heat}P_{arc}dt$$ (5)

The heat transfer coefficient *k*_*heat*_ determines the fraction of the arc energy which contributes to the gas internal pressure rise. The typical value for *k*_*heat*_ is 22% from the value ranging from 15~45% [12]. *P*_*arc*_ (kW) is the arc power.

According to [12], the volume fraction of hydrogen, acetylene, methane and ethylene in mixed gas are 70%, 15%, 10% and 5% respectively. Besides, with the accumulation of *W*_*heat*_, the gas internal temperature *T*_*gas*_ rises up constantly. This dynamic heating process at constant volume can be expressed as the following form:

$$dQ_{gas}=dW_{heat}=C_{v}m_{gas}dT_{gas}$$ (6)

where *Q*_*gas*_ (kJ) is the quantity of heat; *C*_*v*_ is the specific heat at constant volume of gas mixture, estimated to be 2.84kJ/kg·K; *m*_*gas*_ (kg) is the gas mass.

The internal pressure of the gas is determined using the perfect gas equation. Its differential form is:

$$V_{gas}dp_{gas}=m_{gas}RdT_{gas}$$ (7)

where *p*_*gas*_ (kPa) is the gas internal pressure; *V*_*gas*_ (m^3^) is the gas volume; the gas constant *R* is equal to 1.002 kJ/kg·K for the molar mass of gas mixture of 8.3 g/mol.

Bringing (5), (6) into (7), it can be obtained the relationship between arc energy and corresponding gas internal pressure at average volume ${\bar{V}}_{gas}$:

$$dp_{gas}=\frac{k_{heat}(\gamma -1)}{{\bar{V}}_{gas}}P_{arc}dt$$ (8)

where *γ* is the specific heat capacity ratio of 1.364 for the gas mixture in this paper.

Correspondingly, the change in temperature during arc duration can also be calculated as

$$dT=\frac{k_{heat}}{m_{gas}C_{v}}P_{arc}dt$$ (9)

A logarithmic expression was proposed by SERGI [17,18], and two experimental test campaigns of 62 tests had been carried out from 2002 to 2004 in France and Brazil. Their investigation demonstrated that with the accumulation of arc energy, the gas volume generated increases more and more slowly, which can be approximately regarded as a process of the pressurization at constant volume. In our work, the logarithmic function relation is used in this paper considering that its error is acceptable:

$$V_{gas}=0.44\text{ln}\left(W_{arc}+5474.3\right)-3.8$$ (10)

As the aforementioned analysis, the surrounding liquid inertia prevents the expansion of gas bubble, the pressure difference Δ*p* (kPa) on the interface is given as

$$\Delta p=p_{gas}-p_{0}-p_{oil}-2\sigma_{oil}/r_{gas}$$ (11)

where *p*_*oil*_ (kPa) is the oil pressure at fault location; *σ*_*oil*_/*r*_*gas*_ is the ratio between the surface tension coefficient of the decomposed gas and its radius of curvature and the surface tension coefficient is evaluated at 27×10^−3^ N/m.

The propagation and superposition of the pressure waves, which is generated by internal arcing fault, plays a significant role in tank rupture. Taking into account the attenuation of pressure wave propagation in the insulating oil, the transient pressure acoustic governing equation is given as [19]

$$\frac{1}{\rho c^{2}}\frac{\partial^{2}p_{t}}{\partial t^{2}}-\nabla \left[\frac{\nabla p_{t}}{\rho }-q-\frac{1}{\rho c^{2}}\left(\frac{4\mu }{3}+\mu_{B}\right)\frac{\partial \nabla p_{t}}{\partial t}\right]=Q$$ (12)

where *ρ* (kg/m^3^) refers to the medium density; *c* (m/s) denotes the speed of pressure wave propagation; *ρc* ^2^ is called bulk modulus; *t* (s) is time; *p*_*t*_ (kPa) is the pressure field; **q** is the dipole source (N/m^3^); Q is monopole source (1/s^2^); the dynamic viscosity *μ*and the bulk viscosity *μ*_*B*_ are 5.3×10^−3^ Pa·s and 0.2 Pa·s respectively. The pressure field *p*_*t*_ is the sum of the background pressure field *p*_*b*_ (kPa) and the pressure difference Δ*p* (kPa).

### Tank Wall Mechanical Model

The overpressure due to high-energy arcing faults usually leads to mechanical stress and deformation to transformer tank. The mechanical field characteristics are governed by the three governing equations, which are the transient equilibrium equation, the constitution equation and the compatibility equation, respectively [20]. The transient equilibrium equation (load-stress) with the structural inertia introduced is expressed as

$$\rho_{t}\frac{\partial^{2}u}{\partial t^{2}}-\nabla \cdot \sigma_{t}=F_{v}$$ (13)

where *ρ*_*t*_ (kg/m^3^) is the material density; ***u*** (m) is the displacement of the infinitesimal; *σ*_*t*_ (N/m^2^) is the stress tensor, ***F***_v_ (N/m^3^) is the total load. Its value equals to the ratio between the overpressure *p*_*s*_ (Pa) acting on tank wall and the thickness *d* (m) of tank wall.

According to the Duhamel-Hooke's law, the constitution equation (stress-strain) is expressed as follow:

$$\sigma_{t}-\sigma_{0}=C:\left(\varepsilon_{t}-\varepsilon_{0}-\varepsilon_{p}-\varepsilon_{th}\right)$$ (14)

where *σ*_0_ and *ε*_0_ are initial stress and strain respectively; C is the 4th order elasticity tensor; ':' stands for the double-dot tensor product (or double contraction); *ε*_*p*_ is the plastic strain tensor; the impact of the thermal strain sensor *ε*_*p*_ is ignored.

The total strain tensor *ε* is then written in terms of the displacement ***u*** gradient as the compatibility equation:

$$\varepsilon =\frac{1}{2}\left[\left(\nabla u\right)+{\left(\nabla u\right)}^{T}\right]$$ (15)

The displacement and deformation on the tank under internal overpressure can introduce a structural acceleration based on the second derivatives of the displacements with respect to time. From the acoustics field perspective, this outward-pointing acceleration acting on the boundaries between the solid and the fluid plays a loss role in acoustics pressure boundary [21]:

$$-n\cdot \left[-\frac{1}{\rho }\left(\nabla p_{t}-q\right)\right]=-n\cdot \ddot{u}$$ (16)

where ***n*** is the unit normal vector and ***ü*** is the acceleration.

### Acoustics-mechanical Field Coupling Calculation

**Fig 2:**
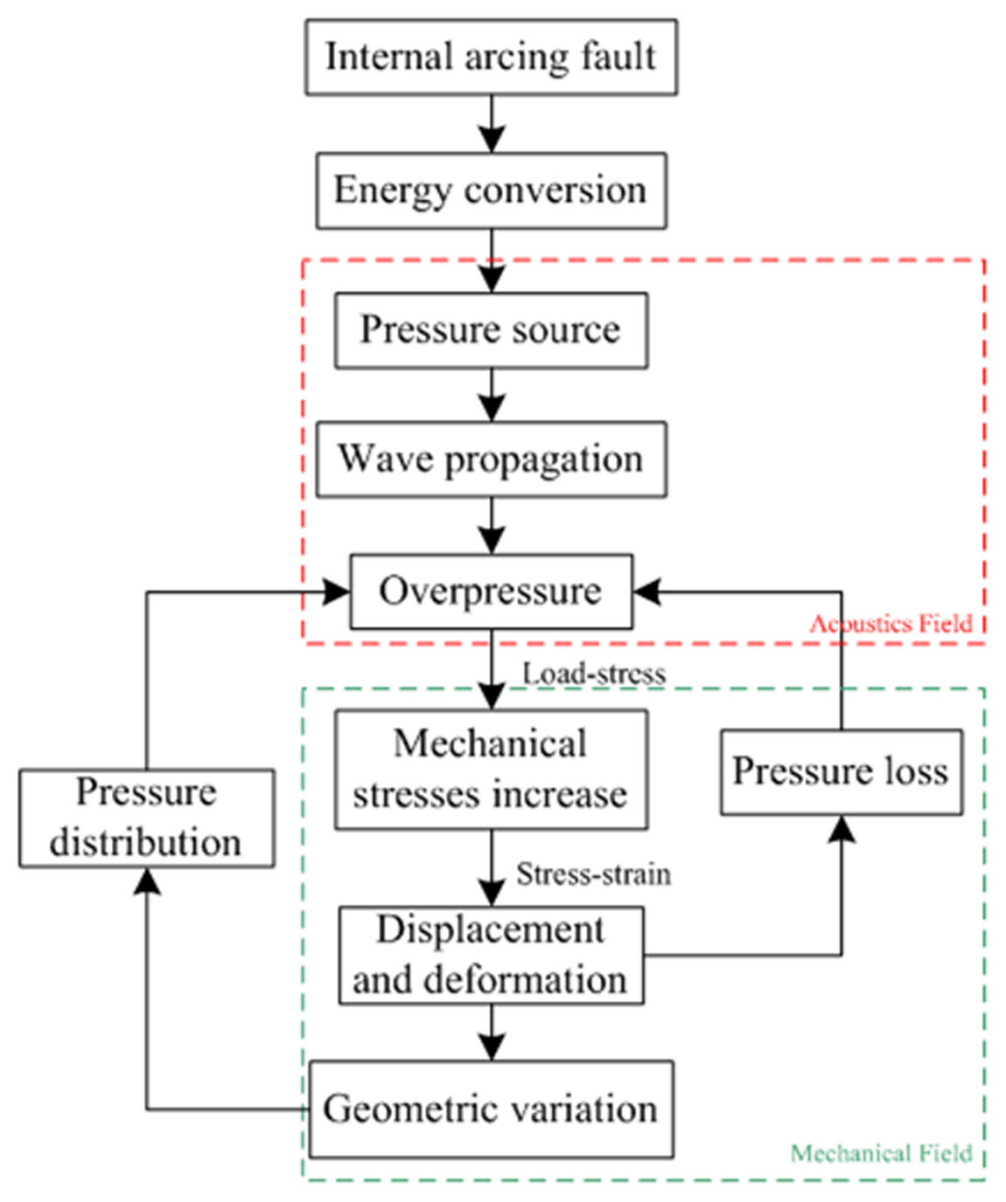
Acoustics-mechanical field coupling relation.

As shown in Fig 2, there is a multi-field coupling relation between the internal overpressure acoustics field and the tank mechanical field. On the one hand, the combined effects of the static overpressure and dynamic pressure wave propagation result in the increase of mechanical stresses that acts on the tank wall, up to rupture sometimes. On the other hand, the displacement and deformation of the tank could create a pressure loss on the boundaries between the tank and the insulating oil in reverse, and the more strain of the tank, the more pressure loss of the acoustics field. Additionally, the geometric variation of the tank wall under the impact from overpressure could influence the wave propagation and change the original pressure field distribution.

### Power Transformer 3-D Simulation Model

#### Transformer Geometric Model

**Fig 3:**
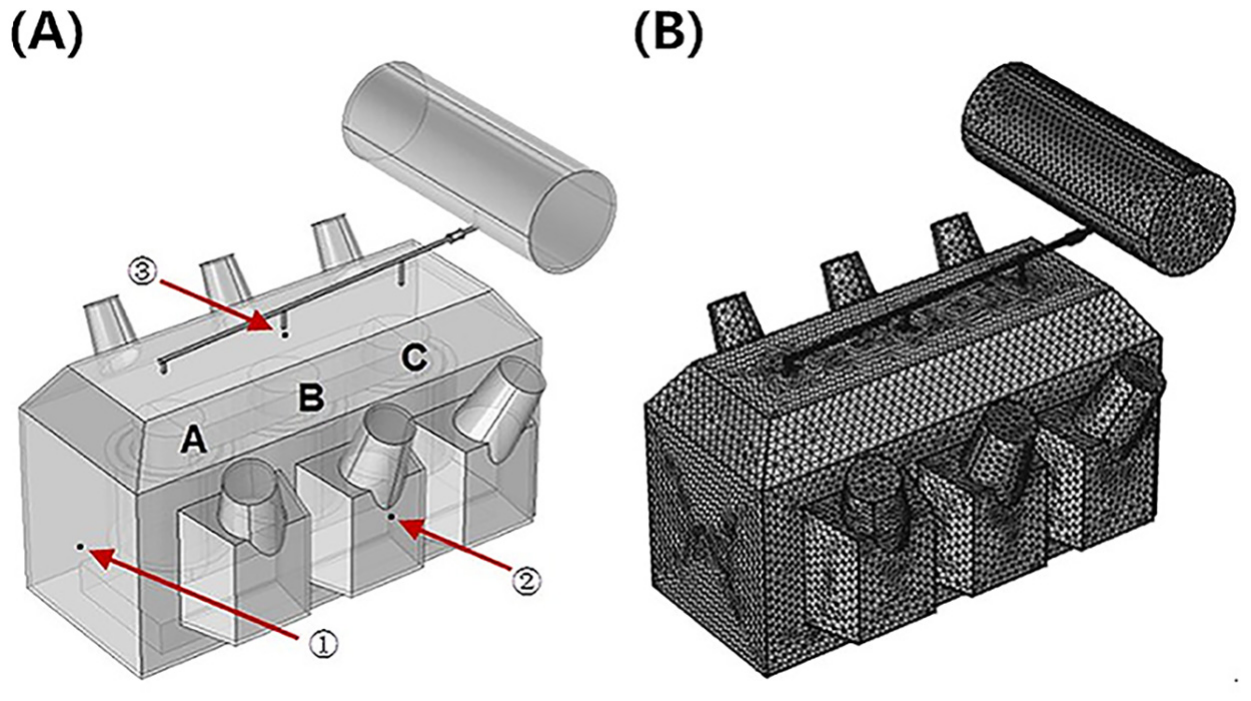
Meshes of simulation transformer tank. (A) Geometric model. (B) Mesh model.

According to the practical geometric and nameplate parameters, a 3-D simulation model for the representative 360 MVA/220 kV power transformer (14 m × 5.6 m × 7.6 m) was built as a study case. The simulation transformer 3D geometric model is provided in Fig 3 below, where the numbers ① ~ ③ refer to three measuring positions, i.e., the broadside, frontal and top faces of the tank, respectively. The letters ‘A’, ‘B’ and ‘C’ refer to the three phases inside the transformer. Since the mesh size and number of elements could significantly influence the calculation time and the accuracy of the simulation results, the mesh model are consisted of 135338 tetrahedral elements, 41730 triangular elements, 3694 edge element and 338 vertex elements. Moreover, some relevant parameters are listed in Table 1 and the acoustic properties of transformer elements involved are summarized in Table 2.

**Table 1:** Geometric and nameplate parameters of 360MVA/220kV power transformer.

Rated capacity	360MVA	Rated frequency	50Hz
Rated voltage	220 kV/15.75 kV	Rated current	945 A/13197 A
No-load current	0.15%	No-load loss	161 kW
Impedance voltage	13%	Load loss	731 kW
Tank length	13980 mm	Tank width	5570 mm
Tank height	7590 mm	Tank wall thickness	12 mm
HV chimney radius	940 mm	LV chimney radius	710 mm
HV chimney height	3160 mm	LV chimney height	2320 mm
Conservator radius	1470 mm	Conservator length	7600 mm
Duct inner diameter	80 mm	Duct length	11260 mm

**Table 2:** The material acoustic properties of transformer elements.

Elements	Material	Density	Speed of sound
Transformer oil	Insulating oil	860 kg/m^3^	1260 m/s
Windings	Copper	8900 kg/m^3^	3810 m/s
Iron core	Soft iron	7660 kg/m^3^	5150 m/s
Air	Air	1.29 kg/m^3^	340 m/s

#### Mechanical Property of Tank wall

**Fig 4:**
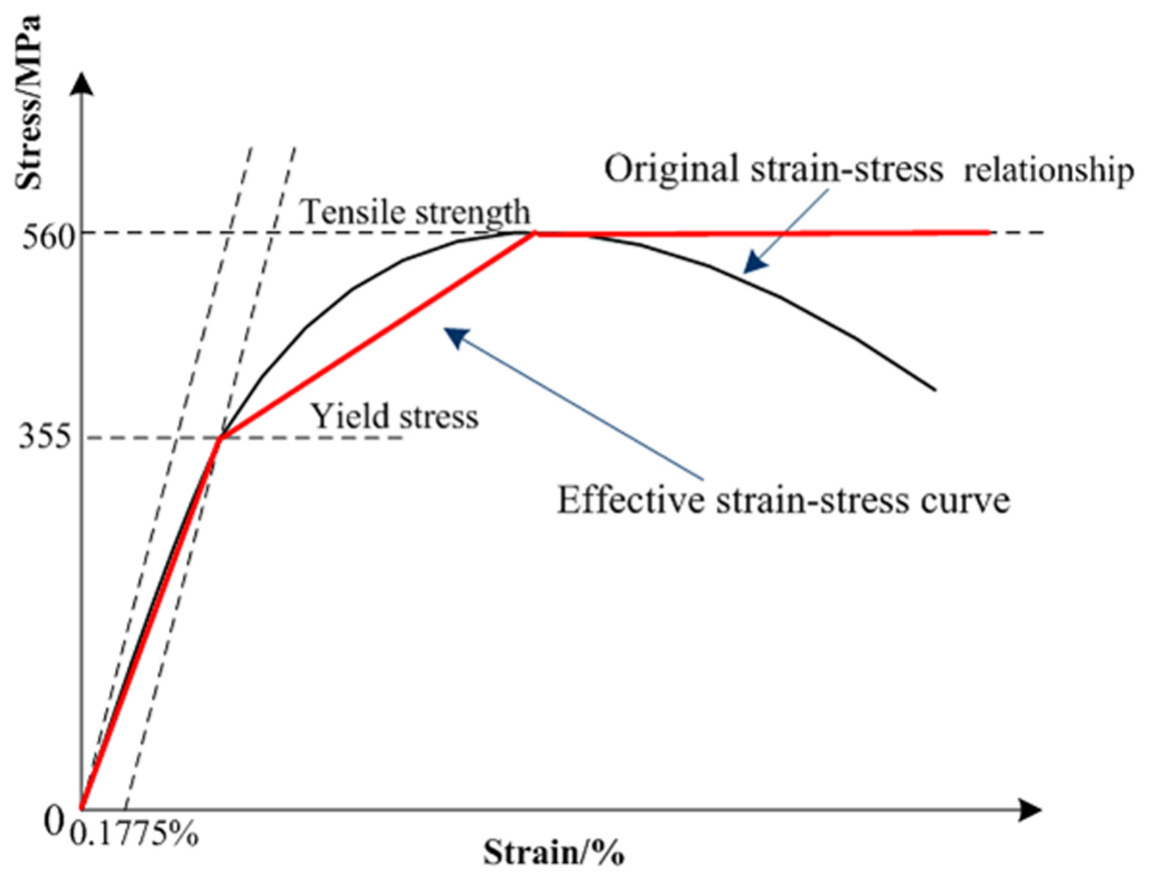
S355J0 carbon structural steel trilinear isotropic hardening model.

The transformer tank is made of S355J0 carbon structural steel and its elastic-plastic behavior is described by a trilinear isotropic hardening model in our work. As shown in Fig 4, the first stage is the linear Young's modules of S355J0 steel up to the yield stress *R*_*p*_ = 355 MPa where the gradient is 200 GPa [22]. In this stage, the material presents elastic deformation without any unrecoverable behavior. When the tank material deforms beyond the strain that elastic deformation persists, the permanent plastic deformation occurs. The tank wall tensile strength is taken as 560 MPa. Once the mechanical stress reaches the tensile strength, the material would be ripped. Additionally, the Poisson ratio of S355J0 carbon structural steel used is 0.3 and its density is 7850 kg/m^3^.

The acoustic and mechanical effects of the arcing faults inside the transformer are calculated numerically using the FEM supported by the acoustic-solid interaction module in COMSOL Multiphysics (version 4.3a) within the transient state [23]. In FEM calculation, the Multi-frontal Massively Parallel sparse direct Solver has been applied for the transient calculation. The time step has been set as 1*μ*s in later three examples according to the Courant-Friedrichs-Lewy criterion proposed in [24]. The simulations were performed on an Inter(R) Xeon(R) E5620, 2.4GHz, eight-cores dual-processors workstation running 64-bit Window 7 and the total calculation time for each example was nearly 116 hours.

## Results and Discussion

**Fig 5:**
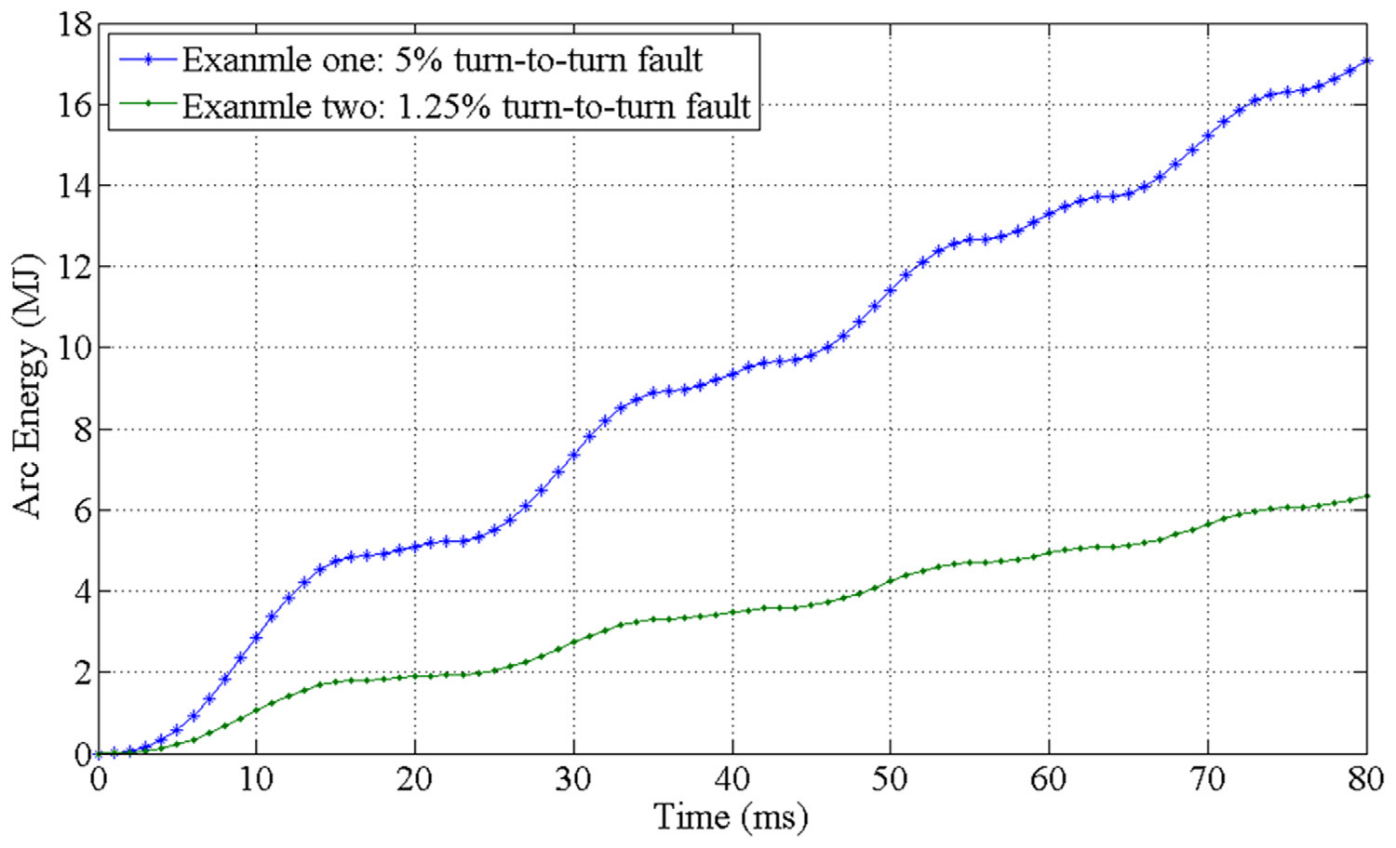
The cumulated arcing fault energy rise curve of both examples.

Power transformer winding turn-to-turn faults are one of the most common and serious fault types. In the simulation tests, a 5% turn-to-turn fault in phase A high-voltage(HV) winding side and a 1.25% turn-to-turn fault in phase C low-voltage(LV) winding side were applied. As shown in Fig 5, the cumulated arc energy curves in the examples are approximately a linear function of the simulation time. In the first example, the 5% inter-turn fault generates more than 17MJ arc energy within 80ms. Similarly, nearly 6.3MJ arc energy was released by 1.25% inter-turn fault in the second example.

### Example One: 5% Turn-to-turn Fault

**Fig 6:**
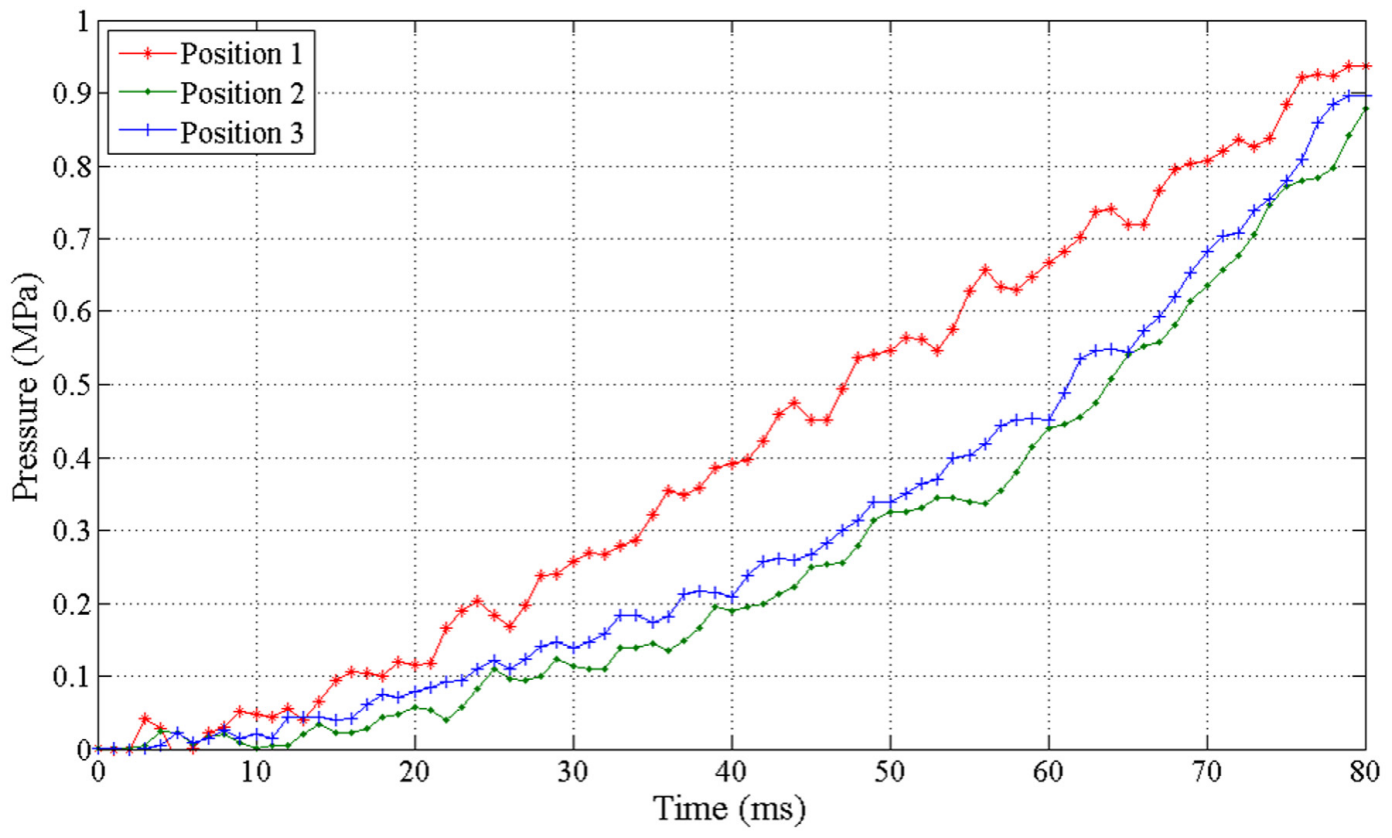
Tank internal overpressure distribution with 17.3MJ arcing fault. (A) t = 20ms. (B) t = 40ms. (C) t = 60ms. (D) t = 80ms.

A 5% turn-to-turn fault was provoked outside the phase A high voltage winding side and the fault location is at 1/3 of the total winding height. The fault occurs at 0 ms, and the arc duration is 80 ms corresponding to the average response time of a traditional circuit breaker. Fig 6 illustrates the transformer tank internal overpressure distribution caused by 17.3MJ arcing fault at 20ms, 40ms, 60ms and 80ms, respectively.

**Fig 7:**
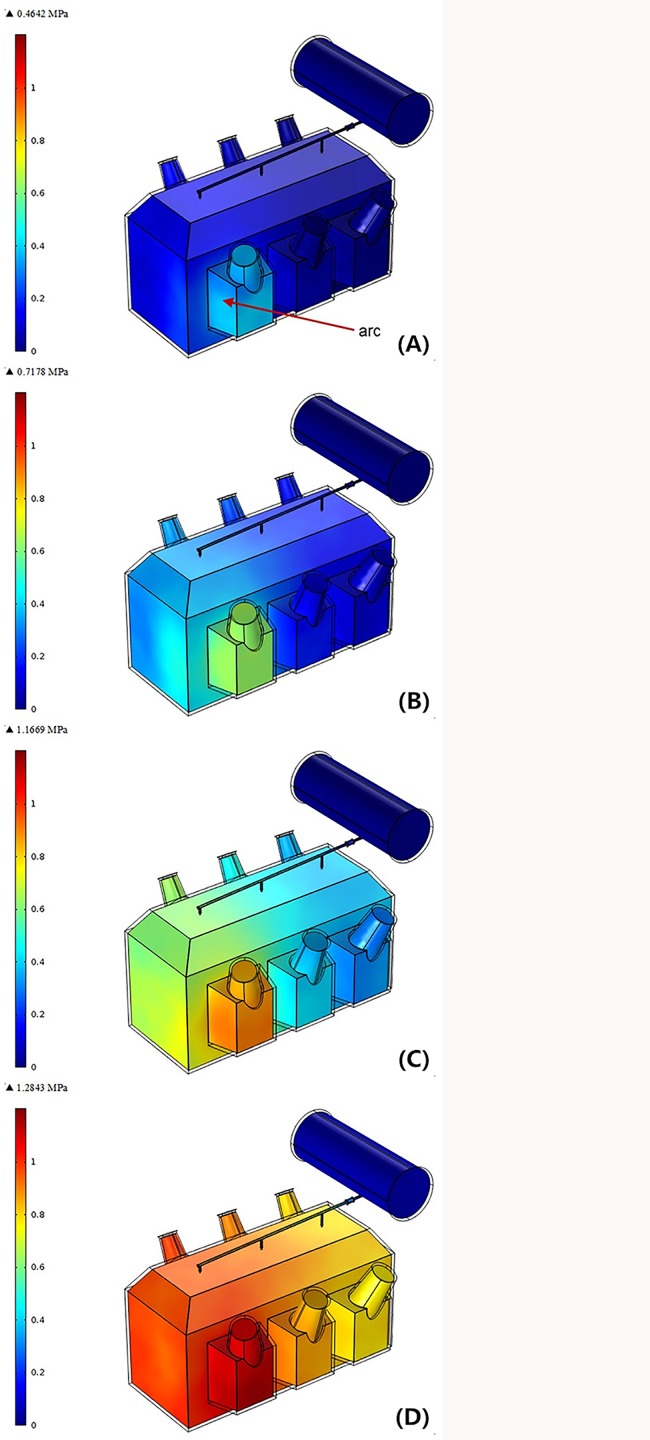
Transformer tank internal overpressure curves at three positions during 80 ms.

Correspondingly, the transformer tank internal overpressure curves at three positions have been plotted in Fig 7.

By analyzing the simulation results about the overpressure characteristics inside tank, some conclusions have been drawn below.

1. As shown in Figs 6 and 7, the pressure level inside tank rises up drastically with the accumulation of arc energy. The maximum transient rate is more than 30MPa/s. When the simulation time evolves to 80ms, the average pressure level at the three locations is greater than 0.9MPa and the maximum inside tank is approximately 1.3MPa, near six times higher than the static withstand limit pressure.
2. It is also found that the pressure is not spatially uniform inside the tank, the positions close to the arc actually has much larger pressure than other regions. This is because the mechanical deformation of the transformer tank has absorbed a certain amount of arc energy during the transfer of pressure wave. The further the position is away from the fault center, the smaller the pressure measures.
3. Since the pressure of fault center varies with the change of arc energy and also considering the impact of metal components and insulation fabrics on reflection of pressure waves, the three internal pressure rise curves corresponding to the three points are different from each other with respect to the frequency of variation on curves though they share some similarities in the increase trend.

**Fig 8:**
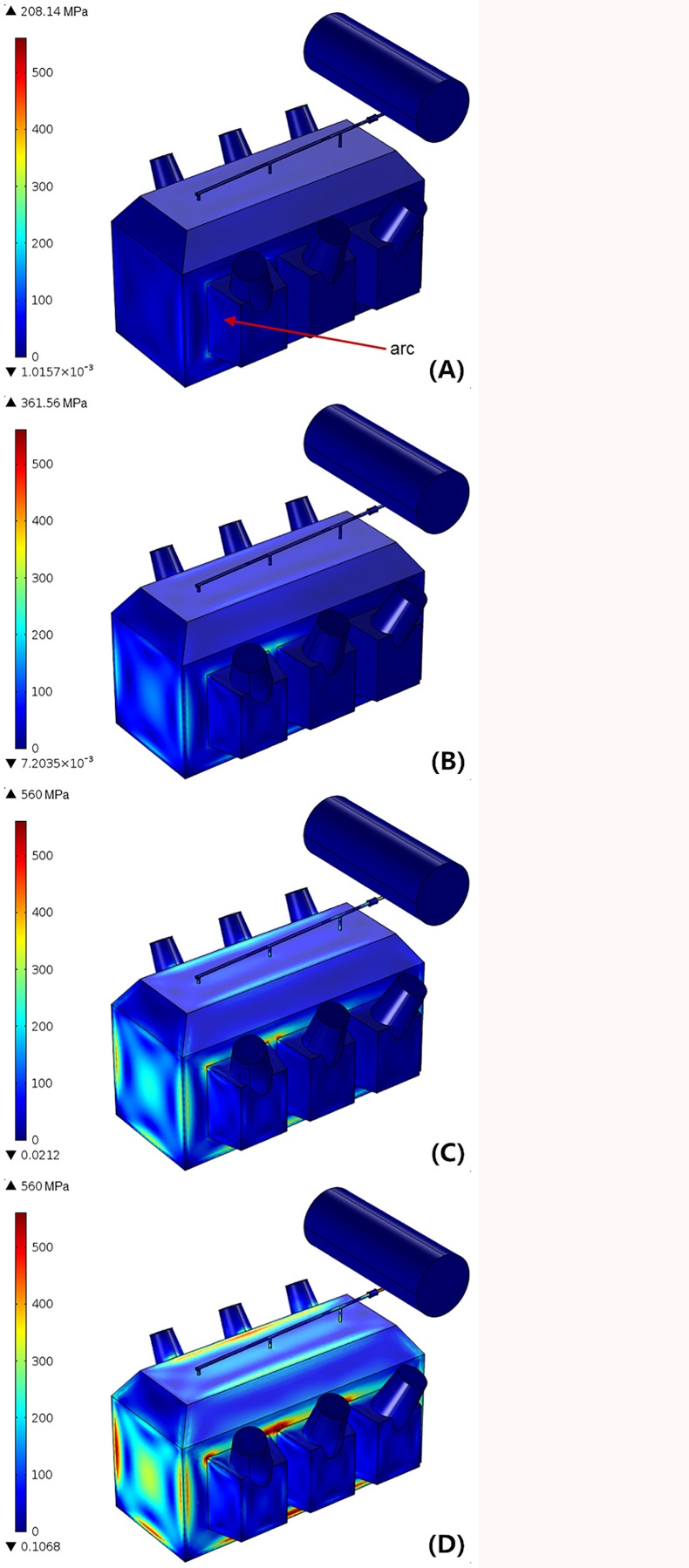
von-Mises stress in transformer tank with 17.3MJ arcing fault. (A) t = 20ms. (B) t = 40ms. (C) t = 60ms. (D) t = 80ms.

To understand the essence of tank deformation or explosion, the tank mechanical behavior under overpressure has been simulated in the test, which is the key factor to determine whether or not the transformer can withstand overpressure caused by high-energy arc without being ruptured. The von-Mises stresses evolution in tank wall over time was plotted in Fig 8.

As shown in Fig 8, we solved the transient mechanical effects of arcing faults within the transformer tank: as the internal pressure varies, the mechanical stress in the tank has a significant increase accordingly. At 40ms after initiation of the fault, the von-Mises stresses have exceeded 360MPa in the joint between frontal face and chimney (close to the fault location), which are less than the tensile strength of steel, but more than the yield stress. It means that these parts of tank have been unrecoverable deformed without being ripped. Furthermore, when the arcing fault duration is longer than 60ms, the von-Mises stresses rise up to 560MPa along the joints on the chimney bottom. This value reaches the tensile strength of steel. As a result, the tank wall has already ruptured.

### Example Two: 1.25% Turn-to-turn Fault

**Fig 9:**
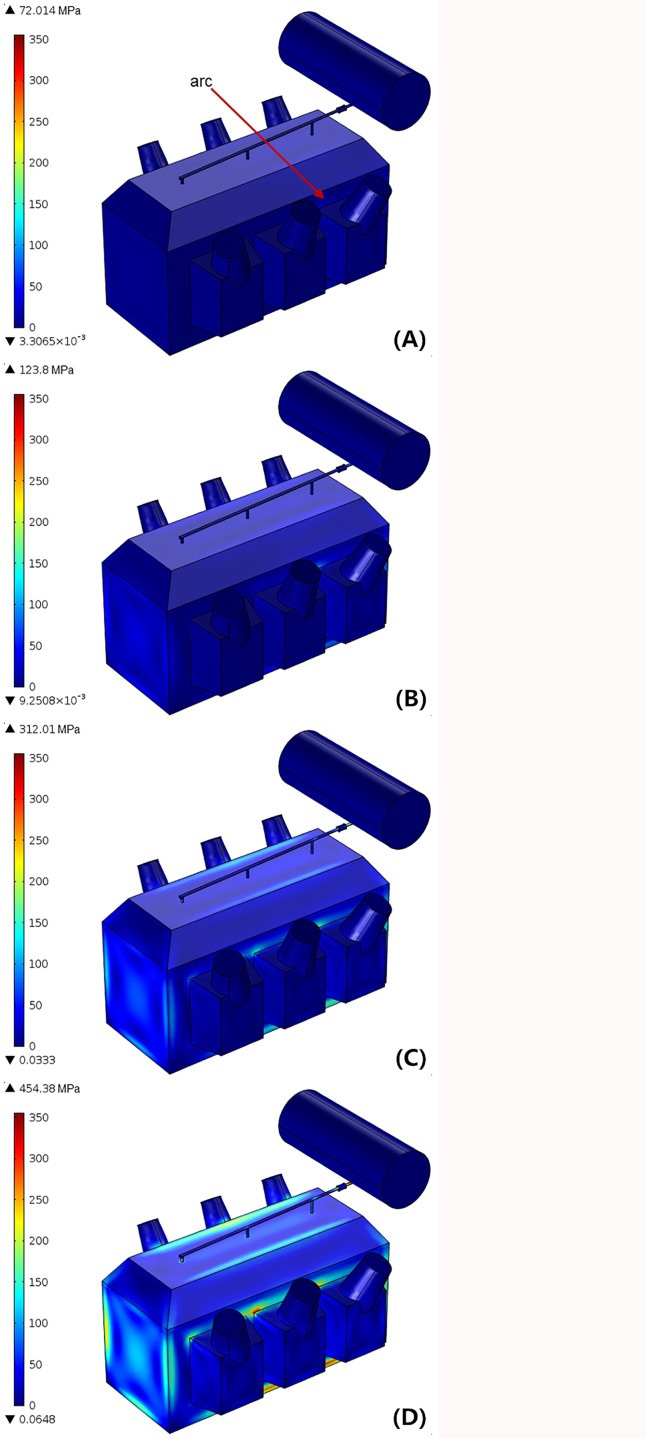
von-Mises stress in transformer tank with 6.3MJ arcing fault. (A) t = 20ms. (B) t = 40ms. (C) t = 60ms. (D) t = 80ms.

A 1.25% turn-to-turn fault was provoked outside the phase C low voltage winding, and the fault location is at 2/3 of the total winding height in example two. With the same parameters and conditions as applied in the above example, the transient mechanical stress distribution in tank wall was given in Fig 9.

In this example, the released arc energy is 6.3 MJ, nearly one-third of the previous example. Since the fault arc is located in phase C between the low voltage winding side and the iron core, the propagation of fault pressure waves is seriously damped by the surrounding metal construction and insulating materials. As demonstrated in Fig 9A–9C, the mechanical stresses in the tank are smaller than 355 MPa (the steel yield stress) during the first 60ms. Namely, when the accumulated arc energy is less than 5 MJ, this type of transformer tank could resist the impact from internal overpressure with the elastic deformation. Then, when the total energy rises, a higher stress concentration, more than 355 MPa but less than 560 MPa, can be seen in some joints at 80 ms. At this value, these local joints in the transformer tank experienced plastic deformation rather than rupture.

### Analysis and Discussion

Previously, both the acoustics and mechanical effects of the internal arcing faults within the same transformer were simulated in the above examples. The fault severity and fault location in these two examples are different from each other. By comparing their results, some conclusions have been drawn below.

1. The simulation examples reveal that when an electrical arcing fault occurs inside transformer tank, a huge volume of explosive gas is created within tens of milliseconds. This huge gas yielded by the fault stimulates pressure wave propagation and overpressure inside tank. Once the superposition of static overpressure and dynamic pressure due to the wave propagation exceed the mechanical strength of the transformer tank, tank deformation or explosion is inevitable. Moreover, it appears from the above two examples that there is a positive correlation between the severity of tank deformation and the arc energy.
2. The arc energy is determined by arc voltage, current and its duration. In general, the magnitude of the arcing fault, especially arc current associated X/R (ratio of reactance to resistance) of the circuit, is uncontrollable. If the faults can be cleared in a timely manner, less energy would be released. In the second example, the von-Mises stresses in tank wall do not exceed the elastic limit in the first 60 ms, though the internal overpressure is more than 0.2 MPa (the maximum operating pressure) at that time. Obviously, if the short circuit fault in this case can be cleared in less than 60 ms, the transformer tank would endure the internal overpressure without any permanent plastic deformation. As mentioned above, the rapid fault clearance is an effective way to control the released arc energy as well as to prevent transformer tank permanent deformation, even explosion.
3. In the aforementioned simulations, there appear some conspicuous stress concentrative points in connecting parts of tank wall, such as the joint at the chimney to the tank wall, joint at the cover to tank wall and joint at the tank wall to the base plate. In order to prevent the tank from unrecoverable deformation or explosion, the mechanical stresses in tank wall, especially in these highest stress parts, must be kept lower than the steel yield stress. In other words, one good way to improve the strength of transformer tank is increasing the mechanical property of the local high stress areas.
4. The inner diameter of the duct connecting tank and conservator is only 80 mm, and its length is more than 11000 mm, which has played a role in damping overpressure wave propagation. As shown in Figs 6–9, the acoustics and mechanical effects of the arcing fault within the conservator are negligible. In fact, the air space in conservator has considerable pressure enduring capability due to its excellent compressibility. Hence an expansion of cross-sectional area of the connection duct or extra pipes with a larger inner diameter which provide the mitigation passageways are recommended to depressurize the magnitude of the overpressure inside the main tank with no delays.

## Conclusions

This paper investigates the mechanical response of tank wall under internal overpressure due to the high energy arc fault in a 360 MVA/220 kV oil-immersed power transformer through a 3D numerical model using finite element analysis. With the proposed model, the dynamic propagation and distribution of the overpressure wave are described. The tank transient deformation and physical rupture, particularly resulting from internal arcing faults, are also explained. The simulation results show that the pressure and mechanical stress increase significantly within a few milliseconds after an internal fault occurs. During this very short time, an approximate proportional relationship exists between the overpressure amplitude and the energy released by the arc fault. The overpressure wave propagates along the tank wall and is prone to exert huge stress on some connecting parts, such as the joint at the chimney to the tank wall, joint at the cover to tank wall and joint at the tank wall to the base plate. The longer the fault lasts, the higher the possibility of occurrence of tank deformation at these points is. Therefore, the quality and condition of these areas of highest stress are critical in determining the capability of transformer tank to withstand the high energy arcing fault without being deformed or ruptured.

More importantly, the theoretical model and simulation method proposed in this paper provide a guidance for fault characteristics analysis and mitigation of oil-immersed transformers, which could be an economic and safe alternative approach of costly and dangerous field tests.

## Supporting Information

S1 Text The 3D dataset of power transformer internal overpressure distribution at 20ms in example one. (ZIP)

S2 Text The 3D dataset of power transformer internal overpressure distribution at 40ms in example one. (ZIP)

S3 Text The 3D dataset of power transformer internal overpressure distribution at 60ms in example one. (ZIP)

S4 Text The 3D dataset of power transformer internal overpressure distribution at 80ms in example one. (ZIP)

S5 Text The 3D dataset of von-Mises stress in transformer tank at 20ms in example one. (ZIP)

S6 Text The 3D dataset of von-Mises stress in transformer tank at 40ms in example one. (ZIP)

S7 Text The 3D dataset of von-Mises stress in transformer tank at 60ms in example one. (ZIP)

S8 Text The 3D dataset of von-Mises stress in transformer tank at 80ms in example one. (ZIP)

S9 Text The 3D dataset of von-Mises stress in transformer tank at 20ms in example two. (ZIP)

S10 Text The 3D dataset of von-Mises stress in transformer tank at 40ms in example two. (ZIP)

S11 Text The 3D dataset of von-Mises stress in transformer tank at 60ms in example two. (ZIP)

S12 Text The 3D dataset of von-Mises stress in transformer tank at 80ms in example two. (ZIP)
