# Correction: Numerical Methods for the Analysis of Power Transformer Tank Deformation and Rupture Due to Internal Arcing Faults

**DOI:** 10.1371/journal.pone.0137038

**Published:** 2015-08-25

**Authors:** Chenguang Yan, Zhiguo Hao, Song Zhang, Baohui Zhang, Tao Zheng

The order of Figs. 6 and 7 is switched. The figure captions appear in the correct order.
